# Explaining time elapsed prior to cancer diagnosis: patients’ perspectives

**DOI:** 10.1186/s12913-017-2390-1

**Published:** 2017-06-28

**Authors:** Astrid Brousselle, Mylaine Breton, Lynda Benhadj, Dominique Tremblay, Sylvie Provost, Danièle Roberge, Raynald Pineault, Pierre Tousignant

**Affiliations:** 1 Département des Sciences de la Santé Communautaire, Centre de recherche - Hôpital Charles-Le Moyne, Université de Sherbrooke, 150 Place Charles LeMoyne bureau 200, Longueuil, Quebec Canada; 2 Université de Sherbrooke, Longueuil, Quebec Canada; 3 École des Sciences Infirmières, Centre de recherche - Hôpital Charles-Le Moyne, Université de Sherbrooke, Longueuil, Quebec Canada; 4 Direction de Santé Publique de Montréal, Institut de Recherche en Santé Publique de l’Université de Montréal, Montreal, Quebec Canada; 5 Direction de Santé Publique de Montréal, McGill University Health Centre, Montreal, Quebec Canada

**Keywords:** Cancer, Early diagnosis, Primary care

## Abstract

**Background:**

Cancer is the leading cause of death in Canada. Early cancer diagnosis could improve patients’ prognosis and quality of life. This study aimed to analyze the factors influencing elapsed time between the first help-seeking trigger and cancer diagnosis with respect to the three most common and deadliest cancer types: lung, breast, and colorectal.

**Methods:**

This paper presents the qualitative component of a larger project based on a sequential explanatory design. Twenty-two patients diagnosed were interviewed, between 2011 to 2013, in oncology clinics of four hospitals in the two most populous regions in Quebec (Canada). Transcripts were analyzed using the Model of Pathways to Treatment.

**Results:**

Pre-diagnosis elapsed time and phases are difficult to appraise precisely and vary according to cancer sites and symptoms specificity. This observation makes the Model of Pathways to Treatment challenging to use to analyze patients’ experiences. Analyses identified factors contributing to elapsed time that are linked to type of cancer, to patients, and to health system organization.

**Conclusions:**

This research allowed us to identify avenues for reducing the intervals between first symptoms and cancer diagnosis. The existence of inequities in access to diagnostic services, even in a universal healthcare system, was highlighted.

## Background

Cancer is the primary cause of death in Canada (30% of deaths), ahead of cardiovascular and respiratory diseases [1]. Two out of four Canadians will develop cancer, and one Canadian in four will die from it. More than half of newly-diagnosed cancers are lung, breast, colorectal, or prostate cancers. The most fatal cancers are also the most common: lung cancer accounts for around 27% of cancer deaths, colorectal cancer causes 11.5% of cancer deaths in women and nearly 13% in men, and 14% of cancer deaths among women are due to breast cancer [1].

The precise impact on cancer progression or survival of the interval between first symptoms and cancer diagnosis is difficult to estimate [2–5]. However, shortening this interval could lead to diagnosis at earlier stages of illness, thereby improving patient prognosis [2, 6, 7] and quality of life post-treatment [8], especially in cases of breast and colorectal cancer [2]. Furthermore, the period from first signs to diagnosis appears to be a key determinant of cancer outcomes [7]. Gaining a better understanding of what happens in the time preceding cancer diagnosis will provide a clearer picture of the current situation and help identify levers for improving patients’ pre-diagnostic pathways [9]. Writings suggest that delays before diagnosis are related to various factors that can refer to the characteristics of cancer, to the patient, and to the healthcare system [7, 10–12]. In this study’s context, in which patients experience recurrent problems in accessing primary care and diagnostic services [13], it is important to document and understand the factors explaining such delays.

The objective of this article is to analyze the factors influencing time elapsed between the first help-seeking trigger—such as the first appearance of symptoms or a letter of invitation to take part in a provincial cancer screening program—and cancer diagnosis. The study looked at the three cancer types that are most common and have the highest mortality rates: lung, breast, and colorectal. This was a qualitative analysis based on patients’ experiences and perspectives. This study was conducted in Quebec, which has a free universal healthcare system in which primary care, diagnostic, and specialized services are mainly covered by public funding. Patients may also, if they wish and can afford it, use certain diagnostic services provided in private clinics. The results of this study will be of interest to health services researchers, policy-makers, managers, and practitioners involved in optimizing health system organization, particularly with respect to cancer, primary care, and diagnostic services.

### New contribution

The contribution of this paper is threefold. First, it revisits the Model of Pathways to Treatment, the current reference for analyzing intervals in early cancer diagnosis. In particular, the blurred boundaries between the first stages of appraisal and help-seeking, the non-linear patient pathways in these stages, and the fact that phases vary depending on the type of cancer make time intervals as identified in the Model of Pathways to Treatment difficult to operationalize. Other authors have successfully used this model to estimate time intervals [14]. However, these studies do not analyze first stages, which are usually not covered by administrative data sets, which might explain why the Model of Pathways to Treatment was considered to be really helpful. Second, the present paper highlights the importance of primary care organization and coordination for shortening time elapsed before diagnosis. Third, it reveals the existence of significant access inequities even in a universal and public healthcare system.

## Methods

### Conceptual framework

**Fig. 1:**
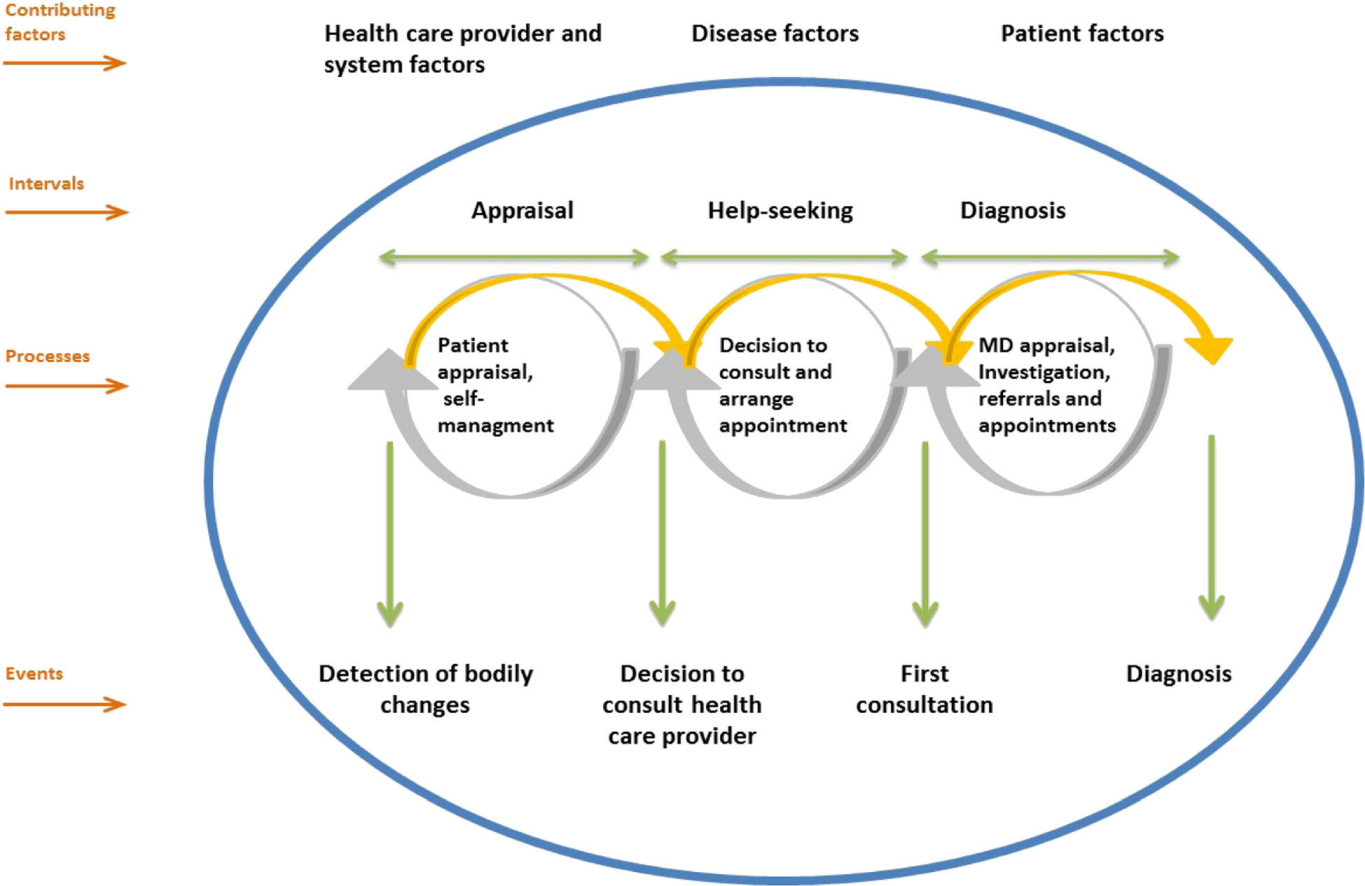
The Model of Pathways to Treatment

The model around which there appears to be consensus on the conceptual representation of intervals and their determinants, of processes, and of events related to care access is the Model of Pathways to Treatment, referred to above [7, 10]. This model, a refinement of Andersen’s model [8, 15], identifies four time intervals: 1) The “appraisal interval” is the period in which the person notices somatic changes, solicits others’ opinions, and looks for ways to minimize symptoms, such as through self-medication or lifestyle modifications. 2) The “help-seeking interval” stretches from the moment when the person decides on the need to consult up to the first visit with a physician. 3) The “diagnostic interval” is the period of investigation, during which the physician documents symptoms and prescribes various tests and examinations. If the physician’s diagnosis is inconclusive, the patient reverts back to the “appraisal interval” [10]. 4) Once a diagnosis has been made, there is a “pre-treatment interval”, during which the treatment is planned. Our study covers the first three stages, as it is focused on time elapsed to diagnosis. The conceptual framework is presented in Fig. 1. It is an adaptation of the Model of Pathways to Treatment [7, 10], as our study does not cover the post cancer diagnosis period.

The time elapsed before diagnosis varies depending on factors related to the characteristics of cancer, to the patient, and to the healthcare system [7, 10–12]. Among the factors related to cancer characteristics are symptom non-specificity [16, 17], symptom acuity [11], and presence of pain [11].

Among the personal factors related to patients are: advanced age [18–20]; family history [18, 21]; sex [11]; low education level and social environment [18, 21, 22]; social isolation [11, 18, 23]; presence of comorbidities [11, 24, 25]; attitude toward symptoms [11, 26], such as minimization [27], fear of diagnosis [11, 28, 29], and feelings about diagnostic testing (embarrassment, fear of pain) [29–31]; patient’s psychological status [32, 33]; postponement of appointments [34]; low frequency of medical visits [17, 21, 35, 36]; ethnicity [11, 37]; and socioeconomically disadvantaged status [11].

Factors related to health system organization include difficult access to primary care, diagnostic, and specialized services [38–40]; lack of medical insurance [41, 42]; lack of a family physician [43]; size of wait lists [32]; physician behaviour and practice routines [44]; physician–patient communication [45, 46]; discriminatory practices [11, 23]; symptom-recognition skills; ability to interpret tests; quality of referrals; systematic screening [11, 47, 48]; constraints related to the institution [31, 34, 49]; availability of materials and personnel; and the organization’s curative vs. preventive priorities [50].

### Study design and collection and analysis of qualitative data

The analyses presented here were nested in a larger project based on a sequential explanatory design [51] that was aimed at understanding the role of primary care clinic affiliation in early cancer diagnosis. That project involved two complementary data collection strategies. First, a quantitative study was conducted among 438 adults with cancer (breast, lung, or colorectal) enrolled for less than three months in an oncology clinic in one of the four participating hospitals located in Quebec’s two most populated regions. If eligible for the study, patients were invited by nurses to participate in the study (methodological details regarding the survey are available in Provost et al., 2015) [9]. Time to cancer diagnosis was estimated based on dates provided by the patient: “date when the patient began to have unusual symptoms or signs that could now be attributed to the cancer; date when the patient made an appointment with a physician for these unusual signs or symptoms; date when the physician prescribed tests to diagnose the cancer; and date when the patient received the cancer diagnosis” [9]. When patients were unable to provide an exact date, they were asked for information on the month and year of the event. We used oncology clinics’ cancer registries to estimate residual missing data. Second, a qualitative study was conducted to explain variations in time elapsed between first symptoms and diagnosis, in order to better understand, from the patients’ perspective, how these intervals were influenced by personal, cancer-related, and primary care characteristics. We conducted 22 in-depth interviews, between the summer of 2011 and the summer of 2012, with patients who had agreed to answer the questionnaire. These patients were purposefully selected to obtain contrasting cases [52] in terms of time elapsed between first symptoms and cancer diagnosis. We identified patients for whom that time was either particularly short or long. We also took into account sex, presence or non-presence of symptoms, and affiliation or non-affiliation with a usual source of care. As the recruitment of survey participants took longer than expected, we began selecting patients for interviews when around two-thirds of our targeted sample had been recruited. We regularly monitored new questionnaires in order to select new potential participants for the qualitative phase of the study. We tried to keep the time between the questionnaire and the interview as short as possible because of the patient’s illness and to minimize memory bias. The participants in our study had been diagnosed with breast (*n* = 7), lung (*n* = 8), or colorectal (*n* = 7) cancer. Our sample consisted of five males and 17 females aged between 46 and 76 years (mean = 60.2; SD = 7.8). Participants were contacted through a letter of invitation. A phone contact was proposed, and the consent form was sent in advance. None of the persons contacted refused to participate. Participants were met in person at the time and place of their convenience, and some chose to be accompanied by a loved one. The consent form was signed before interviews, but the interviewer discussed the form again at the time of the interview to obtain oral consent from participants before proceeding.

Semi-structured interviews lasting approximately one hour were conducted by the team’s research assistants. Open-ended questions probed respondents’ medical consultation habits, their care pathway prior to diagnosis, their attitudes and those of their loved ones, as well as key markers in their care experience over that period. All interviews were audio-recorded with the participants’ consent and subsequently transcribed. All participants signed an information and consent form before the interview in which they were informed that the data collected would be used for scientific purposes in scientific publications.

The project received ethical approval from the four hospitals and from the ethics committee of the research centre where the principal investigator was primarily affiliated.

Two researchers double-coded each interview. All divergences were discussed by the team to obtain a consensual reading. The analysis by cancer type was carried out in three stages. First, data on participants’ age and sex, presence or non-presence of symptoms, affiliation or non-affiliation with a usual source of primary care, and diagnostic tests prescribed were compiled in a table for each type of cancer. A histogram was created showing the intervals of elapsed time experienced by the patients. All intervals were coded separately by the two researchers. Any divergences were discussed in team meetings to reach agreement. Second, each patient’s pathway was summarized. The summaries were then ordered by intervals, from shortest to longest. For each cancer type, we identified the factors that explained the short or long intervals experienced by the patients. Third, the results of the analyses for each of the three cancers were compared to uncover the explanatory factors they had in common and those that differed by cancer type.

It is difficult to say anything conclusive about information saturation. In the analyses, the same types of explanatory factors emerged from one case to the next. In that respect, it could be said we achieved a level of saturation in the analysis. However, because the stories were all very different from each other, we cannot say we explored all possible examples.

## Results

Very early on, it became clear that dividing patients’ experience into time intervals as proposed in the Model of Pathways to Treatment was a challenging methodology to apply to analyzing the interviews. In fact, these intervals represent psychological and behavioural stages, from the standpoint of personal experience. As such, it becomes difficult to draw precise boundaries between the stages and to interpret the care pathways. This model assumes that symptoms and events suggestive of cancer are easily recognizable, which is not always the case. We saw in the transcripts that, when patients recounted their stories, it was sometimes difficult to attribute their symptoms to cancer, and the transition from appraisal to help-seeking, and even sometimes to diagnosis, could be vague and non-linear. Even though Walter et al. (2012) recognize that patients may not experience “a linear passage through these intervals” ((7), p.116) and use double arrows to represent the complexity of pathways, nevertheless the various stages, even if they are real, are sometimes difficult to operationalize in order to analyze time intervals and their determinants in the cancer pre-diagnosis period. Furthermore, as noted by Walter et al. [7], how stages are experienced varies according to cancer sites. Considering all these difficulties, in this study, we ultimately decided to treat the intervals in different ways based on cancer type.

### Lung cancer

Table 1 summarizes the patients’ pathways to illustrate their representations of the period before their cancer diagnosis. As the trajectories are varied and the distinctions between stages can be blurred, we chose to present these in a table rather than inserting verbatim in the narration to give a better sense of each patients’ experience before their cancer diagnosis.

**Table 1:** Patient pathways before diagnosis of lung cancer

#	Interval	From first symptoms to diagnosis
8	2 weeks	An X-ray was ordered for this patient upon consultation with a physiatrist for tendinitis of the arm: “Actually… It was odd. I mean, because it was a long time that I’d been having… that I was aware of symptoms. But I just attributed it to fatigue.” (p. 4) “I had terrible pain in my back, but since it was in my back, I didn’t think it had anything to do with my chest. But in fact, it was because there was a tumour just opposite it… and I was… I was getting out of breath, I couldn’t go very far, but you know, I had things I needed to get done. I just put it all down to fatigue. So I told myself that if I just rested, everything would be okay… Meanwhile… I had a pretty bad tendinitis in my left arm. I was referred to a physiatrist because, you know, well. And then he started looking me over. It’s coming back to me… I don’t know how… He looked at me and he said… Maybe I had on a sweater that was a bit more [inaudible 0:04:54.8]. He said: ‘On that side, there, it’s bigger than on the other side.’ And then, he had a little lamp, and he said ‘I see a shadow.’ I didn’t understand! Then he said, ‘I think…’ He said, ‘Has it been a long time since you’ve had an X-ray?’ ‘Ah’, I said, ‘it’s been at least… 3 or 4 years.’ He said, ‘If I were you, I’d go right away. It’s worrisome.’” (44, p. 4) Soon after that, in a private radiology clinic, the patient had an X-ray whose results were positive. The results were transmitted the next day and the radiologist prescribed a scan. The patient managed to accelerate the transmission of the scan results to her family physician.
6	1.5 months	This person suffered from restless leg syndrome and very quickly her symptoms worsened to the point that she had difficulty sleeping. Following a worsening of her symptoms, she went to the emergency room of a hospital where she had a contact. She was diagnosed with lung cancer the next day, after being admitted to hospital. “And then, my daughter-in-law had a friend who was an emergency physician at the hospital. So my husband called her. He called her, and [name of daughter-in-law], and he said, ‘You need to try, to see if your friend could… » (p. 6) “So then, I went to see her (at the ER),… and on the 14th, they sent me to neurology, and four or five people came to see me, and they told me I had two cancers: one in the brain, and one in the lungs. Then I called my husband.”
4	2 months	This person, very athletic and active, experienced respiratory problems related to activity. “Then, at a certain point, a Sunday, after the holidays, I had a cough that was dry, dry, dry. I’d been having frequent dry coughs, so much that… I said, I’m choking… on my saliva, you know. What’s wrong with me? A dry, dry, dry cough… And one that had been going on for a long time, and had irritated my throat, and I had spit up some blood. Then I said, Oh! What’s [inaudible 0:07:39.6] you know, ah, either it’s… But me, I hadn’t thought about bronchial tubes. I was thinking more that I had irritated my respiratory tract, anyway. I put it out of my mind, to some extent. It had scared me a little, but one of my friends said, ‘No, no, you need to see a doctor, now, really,…’ ‘OK, I will.’ But then January came along… We went for a hike on the mountain, and as I was climbing the stairs, I was, like…. Me, I described it sort of like bronchospasms. All of a sudden, I couldn’t breathe, I was out of breath, air wasn’t getting through there. […] I called that week, I think, for an appointment with my doctor and got one….” (261, p. 7) She contacted her family physician for her activity-related respiratory problems. The patient, a nurse, thought it was asthma. In addition to prescribing inhalers, her family physician ordered an X-ray and referred her for to a respirologist. After an initial positive X-ray, the physician thought it was early pneumonia and prescribed antibiotic treatment and another lung X-ray. After a second positive result, more advanced investigations were undertaken: a scan and bronchoscopy.
5	3 months	Two or three months before her diagnosis, this person had a first case of bronchitis, which was treated. At that time she had a lung X-ray, which was normal (October 2011). She had bronchitis again a few weeks later. Her family physician ordered a second X-ray. Following those results, he referred her to the hospital for further evaluation.
7	3.5 months	This person felt a lump when washing herself. After several months, seeing that the lump was not going away, she tried making an appointment with her family physician. Because the appointment she was given was two to three weeks away, she went to a walk-in clinic. The physician there sent her immediately for X-rays, read the results the same day, and ordered a scan, which the patient underwent two or three days later. Following the scan results, the physician ordered a biopsy and referred her to a respirologist.
1	4 months	This person experienced symptoms of shortness of breath in November 2012. She had three mechanical valves, one of which habitually leaked. She thought her shortness of breath was related to a problem with her valve. She had a scheduled appointment with her family physician in December. She told him about the problem. Her physician referred her to a surgeon, who saw a hematoma. He gave her an appointment for March 14. After the consultation, the surgeon referred her to the emergency cardiology service (swollen hematoma + patient turning blue). The patient underwent a scan and an MRI. The physician told her the problem was not related to her valves and sent her to the emergency room at the general hospital. At the ER, on March 18, the physicians suspected cancer. The patient was hospitalized. She then underwent several tests and surgeries (four bronchoscopies, a biopsy, a mediastinoscopy) before receiving a diagnosis on May 10.
3	4 months	This person said she experienced shortness of breath (in May). She consulted at a nearby clinic. Her physician sent her for a lung X-ray. Even though the radiologist indicated that it was urgent that the patient be referred for further investigation, the physician did not notify the patient. It was only at a later consultation, for acute respiratory problems, that the results of the diagnostic X-ray conducted several months earlier were read and communicated to the patient. The patient then went immediately to the emergency room, and two days after going to the ER, she received a cancer diagnosis.
2	5 months	This person experienced unusual shortness of breath in the autumn. After returning from a trip she had taken over the holidays, she made an appointment with a physician. On January 15 a physician accepted her on his patient roster. The patient underwent an X-ray and blood tests. Her physician referred her to a respirologist, whom she saw at the end of February. That specialist noted the presence of a mass and prescribed a bronchoscopy and pulmonary function testing. The diagnosis was reached on March 7th. The interval between first symptoms and making an appointment with a physician was rather long. Once the patient was in consultation, the time to diagnosis was between one and a half and two months.

### Intervals

The duration of the intervals ranged from two weeks to five months (see Table 1). It was sometimes difficult for patients to quantify the pre-diagnosis elapsed time, not knowing whether certain symptoms (restless leg, back pain, chest pain and fatigue) should be associated with lung cancer or what exactly might be considered the first symptoms. For example, Respondent 8 reported a shorter elapsed time between first symptoms and diagnosis (18 months) in the survey questionnaire than in the interview, during which she said she had been noticing her symptoms for more than two years. The interviews showed how difficult it was to pinpoint when the symptoms suggestive of lung cancer actually started.

### Explanatory factors related to lung cancer

We noted that all participants with lung cancer reported having had health problems in the period preceding their diagnosis. Five out of eight (respondents 1, 2, 3, 4, 5) reported having respiratory symptoms (cough, breathlessness), but none suspected cancer. Three people (respondents 3, 4, 5) were first treated for respiratory infections (pneumonia, bronchitis, asthma, acute respiratory illness) before being sent for more extensive investigation. The reported symptoms were often associated with chronic respiratory or muscular disorders, such that there was no pressure for further consultation. However, four of the eight patients interviewed (respondents 2, 3, 4, 6) started the consultation process for an acute condition when symptoms worsened. There is a certain vagueness to the cancer symptoms that blurs the boundaries between the first three stages of the Model of Pathways to Treatment. Maintaining this division leads to pathways being represented in loops rather than linearly in that model.

### Factors related to patients

Certain personal factors influenced the intervals. We observed that patients with strong social networks were encouraged by their family and friends to consult, which could affect the point at which they took the decision to consult. In four cases (respondents 1, 4, 5, 6), the patient or family took a proactive role that shortened the time to first consultation or accelerated the investigation process.

### Factors related to health system organization

Only two patients (respondents 3, 6) went directly to the emergency room; the others either saw their family physician or visited a walk-in clinic. All were able to see a primary care physician rapidly, once they had taken the decision to consult. The interval between this first medical contact and the first radiology exam was usually less than three weeks. The family physicians communicated the results to the patients within about two days of receiving a positive radiology result. Two people (respondents 1 and 3) experienced delays of over a month: in one case, the result was not communicated to the patient; in the other, the symptoms led to extensive investigation of another suspected illness.

### Breast cancer

Table 2 summarizes the pathways of the patients in our study with breast cancer.

**Table 2:** Patient pathways before diagnosis of breast cancer

#	Interval	From first symptoms to diagnosis
15	1.5 months	This person felt a lump on December 4. She worked in a plastic surgery clinic. She pushed to get an appointment with any of the gynecologists at her gynecology clinic right after the appearance of the first symptoms to obtain a mammogram. She saw the gynecologist one week after the first symptoms. The physician ordered all the tests at the same time, including a surgical consult. She underwent all the investigative tests on the same day. She received her diagnosis less than one month after the positive mammogram.
13	4 months	The person felt a lump during breast self-examination. She talked about it with her family. She went to her gynecologist right away for a mammogram and was tested. Her mammogram was not very clear, so the physicians immediately pursued the investigation further. “But that night, I talked about it with my husband and my daughter, because you always feel a bit silly, right, when… You’re always worried about upsetting people. Anyway, for me… that’s how it is. And then they said to me, ‘Listen, don’t hesitate…” (281, p. 7) The patient consulted in the private sector. Because the mammogram results were inconclusive, the radiologist proposed an ultrasound on the same day: “Finally, well, when I went to XXX on June 16… they did a mammogram [inaudible 08: 15]. They didn’t see anything, so the radiologist who saw me said, ‘I’d like to do an ultrasound right away, but there is a cost.’ Of course, I agreed. So, in the ultrasound they saw something. And then, she said, ‘To be sure there isn’t a cancer growing there… I advise that you make an appointment with us for a biopsy.’ So you see, that meant that, that would have been on July 14, and then, on July 14, the biopsy was done and then I left on vacation. I knew that my gynecologist was also on vacation. And when I got back on August 4, I had a call right away [inaudible 08: 53] from my gynecologist’s secretary saying that she would like to see me on the 8th, which was a Monday. And then, on the 8th, she confirmed that it was cancer.” (p. 8) The biopsy confirmed the result. This patient’s diagnosis was confirmed on September 6, two months after her physician ordered the investigative testing.
14	3 months	This person had a mammogram as part of a breast cancer screening program. She had no symptoms, but her mammogram was positive. Her family physician called her 7 to 10 days after the test. She also received a letter from the Screening Program to verify that her family physician had contacted her. Her physician sent her for an ultrasound in the private sector, saying that it would be faster. Her physician received the results and encouraged her to have a biopsy. The patient got her diagnosis a little more than two months after getting the mammogram results.
12	4.5 months	This woman felt a lump in her breast in the summer. As she was eligible for the province’s breast cancer screening program and had moved to a new region, she contacted them to receive a new mammogram invitation letter. The mammogram was done on November 7. One week later, the laboratory telephoned her to convey the results. “I had already felt a little lump, about the size of a grain of rice, say, no more than that. That was in the summer of 2011, but I ignored it for all kinds of reasons: my work, I…, I…, I had five coworkers who had resigned. It’s a very difficult department, complicated, and it’s becoming impossible to recruit people because they’re afraid of going to work there; so I neglected thing so as not to…. In the end, I was all alone.” When the patient received a call informing her of the positive results of her mammogram, she was advised to undergo the usual testing. She refused, because she felt it would take too long, and decided to consult in the private sector, where she got an appointment three days later. The time between the first positive mammogram and the diagnosis was three days. It should be noted that this person is a nurse who had worked 16 years in a hospital oncology service. “I know very well how things work, the wait times, and in fact I’d been able to see, there, how the system had deteriorated over the past 30 years. But I still had some professional contacts in the specialities. If Dr. Y hadn’t been here, I would have gone to see another in the same hospital that I know well. Or another one at xxx hospital, who’s in radio-oncology, but who would have put me in touch with a hematologist-oncologist. In short, I was well connected. So I had certain advantages, I was well connected, I bypassed the wait times that everyone else has to put up with, I imagine, but… well.” (p. 10).
9	6.5 months	There was a history of breast cancer in her sister. This person did not have a family physician but was followed by a gynecologist. The gynecologist ordered a routine mammogram. The patient postponed the test since she did not like the test: “Having a mammogram is no fun. So I had the… I had the paper, so I went a couple of months without… you know, before making the appointment. At one point, I said to myself…” (p. 3) Her gynecologist called her to tell her that her mammogram was positive and ordered a second mammogram. Shortly after the second mammogram, the patient had an ultrasound, which confirmed the presence of a mass. A biopsy was ordered. She requested an appointment. She called three times and waited two months before someone called her back to make an appointment for the biopsy on August 3. The time between the results of the first positive mammogram and her cancer diagnosis was more than four months.
11	8 months	This woman felt a lump, or more of a discomfort in her breast, in June. “In the month of June, I felt a little lump… I would say, a little discomfort, there, in my breast. But, hey, I said: I often have… You know, I had been menstruating for six months. After the… after my… my endometrial ablation, I menstruated for six months, which was normal for an operation like that. And sure, there was something going on in my breast that was bothering me, but I said, hey, it’s my period, it’s my… my hormones, well, you know. Because I have large breasts, it’s… I had gotten used to that over the years. So I didn’t make much of it, except that in June, my husband’s cousin died of breast cancer. Then, it was like a light went on. Then I said, okay [respondent’s name], maybe you need to see a doctor. That was in the month of June. When I got back from travelling, in October or November… because in September, I felt my lump. We were outside the country. Then, I really felt it, and it was… It was starting to be a little painful, my lump. So then, I said, when I get back from this trip, I definitely need to see a doctor. I went to the doctor, and got the diagnosis in the month of… on January 18, 2011.” (001, p. 1) When she tried to make an appointment with her family physician, she was told he was on vacation. She then decided to consult her gynecologist, who ordered a mammogram. The time between the appointment with the gynecologist and the positive mammogram result was about one month. Her gynecologist received the mammogram results on the day of the exam and sent the patient for an ultrasound. The radiology centre where the mammogram was done was in the same building as the gynecologist’s office. After the results, her physician sent her for a biopsy. The time between the positive mammogram and the cancer diagnosis was nearly three months.
10	13 months	There was a family history. This person had a family physician whom she saw regularly and who prescribed an annual mammogram because of the family history. This person had a breast lump since 2006. The positive results of the mammogram were transmitted to her family physician, but no one notified the patient of the positive result. She learned about the result 11 months later in a routine visit to her family physician. “Aside from having a breast lump that didn’t hurt, I was doing everything right, and having mammograms, and all that. That’s what made me so angry in this whole story, it’s that I wasn’t negligent. And even today, a year later, I can’t accept it! I tell myself, it’s not right! There was something somewhere that… There was someone who didn’t do their job.” (230, p. 1) Her physician noted that the positive mammogram had been done almost a year before and encouraged the patient to start the investigative testing quickly. “So then, she was really angry! She said to me, ‘Now, you’re going to have another mammo. Not in six months, not in two months: Now! And that’s when things started to happen. And when I went to the clinic, they didn’t even want to give me an appointment because it was summer. I said to the woman, I said, ‘Madam, I need an appointment for a mammogram.’ She said, ‘Madam, we don’t have any openings now! Call me in September.’ I said, ‘You don’t understand! It’s an emergency.’ ‘Well, what do you want me to tell you?’ (p. 2) Her family encouraged her to consult in the private sector. The patient had to insist to get an appointment for the two exams that had been prescribed. The fact that her ultrasound was done by a physician who was affiliated with the same hospital as the patient accelerated the biopsy appointment. Interviewer: “OK. So you went through the private sector.” Respondent: “Yes, I had to pay but… There, my husband said, ‘Listen’ he said, ‘it’s your health, I think it’s worth it!’ So I went. They did the mammogram and they told me, if we need a clearer image, we’ll call you within 10 days.’ But they didn’t call me. But I had kept the original of my prescription, and on that prescription, my doctor had written ‘mammogram plus ultrasound’. So I called and asked for an ultrasound appointment, and at first the woman didn’t want to give me an appointment! She said, ‘You weren’t called.’ I said, ‘No, you didn’t call me. But my family doctor wants me to have this ultrasound.’ So I got an appointment, and to my surprise, when I went there, on August 24, it wasn’t a technologist, it was a doctor, a radiologist. She started doing the ultrasound, and she said, ‘Oh,’ she said, ‘you’re not made like everyone else, you, Madam!’ I said, ‘What do you mean by that?’ ‘Well, anyway,’ she said, ‘you’re not made like we see in books.’ I said, ‘Yes, and so?’ She said, ‘You have a mass that’s very large and inflamed.’ And she said, ‘That’s not good!’ So there, I started to panic a little, I’ll admit! And then she asked me, what hospital do you usually go to?’ ‘Well,’ I said, ‘My children were born at XXX.’ She said, ‘Great, that’s where I work. I’ll see you tomorrow for a biopsy.’ And there I was, alone, and she said to me, ‘I think it’s cancer.’”(p. 3)

### The intervals

The longest interval between first symptoms and diagnosis was 13 months and the shortest, one month and a half. The interval between first symptoms and mammography ranged from 11 months to under one month. Only one woman (respondent 15) experienced short intervals for both.

### Explanatory factors related to breast cancer

Either the women noticed the symptoms—typically, in the case of breast cancer, a mass—or the cancer was diagnosed through the provincial screening program. As these patients associated the breast mass with possible breast cancer, there was essentially no “appraisal” interval, in contrast to the lung cancer experience. Moreover, the investigation process for a breast mass is able to rule out cancer directly, making the linear pathway relevant (as opposed to the looped pathway for lung cancer).

### Factors related to patients

Longer intervals between first symptoms and mammography were due primarily to personal attitudes. Some patients delayed the examination either because they minimized the symptoms, or they feared or had a prior negative experience of mammography, or simply because their current activities limited their availability (planned vacation, excessive workload, etc.). Some were encouraged by worried family members to go for investigation sooner. Women who were deeply concerned about the mass appeared to have been especially proactive in initiating the screening process and obtaining, in various ways, a requisition for a mammogram.

Shorter times to investigation appeared to be explained by good knowledge of the healthcare system. We had, in our sample, two expert patients who were able to navigate the system, whether public or private, and whose time to investigation was one month or less. One was a nurse (respondent 12) who had worked a long time in oncology, and the other (respondent 15) was a nurse who reported that she maintained her medical record at home.

Being proactive in managing one’s own medical record appeared to result in shorter times to investigation. Patients who were determined to get an appointment sought options that would provide the examination in the shortest time frame, even if it meant paying for it themselves. Of the seven women, four (respondents 10, 12, 13, 14) used private diagnostic services. Even though some private diagnostic tests are covered by the public health insurance plan, four women (respondents 10, 12, 13, 14) had to pay out of pocket at some point in their pathway. This raises questions regarding equity in terms of access to essential diagnostic services.

### Factors related to health system organization

The intervals between mammography and transmission of results appeared quite variable. Some results were communicated on the same day (three of seven: respondents 10, 11, 13), while others took as much as seven to 10 days when transmitted by the patient’s family physician or gynaecologist. We documented two cases of error (respondents 9 and 10), in which the positive result had not been communicated to the patient. In one, the result was noted when the patient returned to the physician’s office a few months later because the mass in her breast was getting bigger and she had never heard from the doctor’s office. In the other, it was only during the patient’s routine visit almost a year later that her gynecologist saw the previous positive results and was at a loss to explain why no one had called her at the time.

The patients with the longest time to investigation were those with sequential care pathways: they made an appointment and underwent testing, the results were then sent to their physician, who prescribed another examination, and then those results were sent again to the physician, who prescribed yet another examination, etc. Delays were incurred by the friction between each of these stages. In contrast, patients who were able to obtain integrated services experienced shorter elapsed times: positive results were transmitted at once and the next examination was set in motion. These shorter intervals appeared to be due to the fact that technical and medical resources were available on the same site and that diagnostic testing was coordinated.

### Colorectal cancer

Table 3 summarizes patients’ perceptions regarding the period before their cancer diagnosis.

**Table 3:** Patient pathways before diagnosis of colon cancer

#	Interval	From first symptoms to diagnosis
22	1 week	This person had been followed for nearly 30 years in gastroenterology for ulcerative colitis and a non-cancerous tumour. During a routine appointment, the gastroenterologist detected an anomaly and performed a biopsy. The diagnosis was announced less than a week later.
18	4 months	After a routine visit, her family physician ordered a biopsy, which was negative. However, after noticing blood in her stools, the person decided to see her doctor again. “The biopsy was in six months. So he said to me, ‘Listen, go with the private system, $250, it will go faster.’ So, of course, you have a gun to your head, so you go… Finally, the results came back negative […] But in January, then… I had bloody stools. So… then, you start looking. So you go to the walk-in place, they treat you like you’re a bull in a china shop, because you’re not one of their clients! […] But you want an appointment because you have bloody stools! So then they tell you that you need an appointment. So then, the guy, he says to you… He points at you, and says to you, ‘Oh, right, in fact, you have…’ Well, yes! ‘Okay, then, you’ll need an appointment with a… a specialist.’ But there aren’t any until August. No, no, April… the month of April! So, there, because you were threatening. So then you go to see him, and he’s a specialist. He points at you and says, ‘You’re right, you’re bleeding.’ No, now wait a minute: that’s three visits, three times wasted, all because I have blood [in my stools]! But still no tests.” Having been offered an appointment in four months for a colonoscopy, and being a French citizen, the person decided to go to France for treatment. There, within a few days, she underwent the necessary tests and was offered surgery.
20	7 months	The person had a family history of cancer and digestive problems. She took steps immediately when she began experiencing abdominal pain with intense fatigue. However, because she also had hormonal problems, her family physician did not order any other tests at her annual check-up. She returned several weeks later to the walk-in clinic, where antibiotics were prescribed. She went back to see her family physician and obtained a referral to a gastroenterologist, but delayed making an appointment, and when she eventually tried to make one, she found the wait time to be unacceptably long. Finally, because of increasingly severe abdominal pain and an abdominal mass, she decided to go to the emergency room. She was hospitalized, and was first diagnosed with severe anemia, then with an intestinal tumour.
16	8 months	The person consulted her family physician after considerable weight loss. Her physician ordered blood tests and sent her to an internal medicine specialist. After consultation, the latter referred her to a gastroenterologist, who ordered a colonoscopy. “She sent me for a test on my stomach, because I had no symptoms! Everything was working well: the stomach, the… the intestines, it was just… The only thing was… the weight loss. So they started with the stomach: everything looked okay. After that, the next thing was to redo the colonoscopy.” It was the wait for that last exam that took the longest (5 months).
19	12 months (symptoms + treatment for other health problems) + 6 months (investigation)	This person had been feeling very tired for several months: “I was always tired and aching all over. So I decided to have blood tests to see what was wrong. They didn’t find anything. Then I asked my doctor to test for diabetes, because I had a family history, and that’s when they diagnosed diabetes.” After several tests, the person was referred to several different specialists: “Then, he said maybe it was a professional burnout. And that maybe it was also depression. He referred me to an endocrinologist and also to a psychiatrist for an evaluation to see if I was depressed. Which I did, and the psychiatrist said I was in a deep depression; but I kept on telling my doctor, all the doctors, or at least the three doctors I was seeing, that I was depressed because I was fatigued, and because that fatigue came from a physical discomfort that I had all the time, in my buttock and thigh. And then, they told me that it was probably the depression, that I had… physical discomfort because of that. That went on until February, when my buttock swelled up like a balloon and I went to the ER.” In the emergency room, the patient received a diagnosis of perianal abscess. In a follow-up visit, the physician detected an anomaly and referred the patient to surgery. A few days later, the surgeon confirmed the anomaly and prescribed a colonoscopy, which was done a few days later and identified the mass.
17	More than one year	Two years before, this person had consulted a physician, who was concerned about her symptoms. He prescribed a colonoscopy, but the wait time was almost a year and the person gave up. She also refused to pay for the test in the private system. However, the symptoms worsened and she went to the emergency room. After several tests, the emergency physician informed her of the diagnosis.
21	Several years of symptoms and 7 months of investigation	This person had experienced sporadic bleeding over at least 10 years. “In my case, it had been going on for a few years already, that I occasionally had bleeding… when I had a bowel movement. But everyone told me it was hemorrhoids.” The last time she saw a physician, it was when she was accompanying her husband to a medical appointment. The professional prescribed a hemorrhoid cream for her. “Then last year, my husband had some blood tests done that he had sent to his doctor […] So that time, I went with him, and I met the doctor. That doctor was actually pretty old. So I explained the whole thing to him. He said, ‘I’ll do a rectal exam, but…’ He told me it was hemorrhoids, but I said, ‘Still, I’d like to check this out further.’ So he did the rectal exam, but he said, ‘See, it’s hemorrhoids, we can feel them. I’ll give you a cream; it will stop.’ So, it wasn’t a problem. The cream definitely helped, and it stopped.” However, a few months later, there was a lot of bleeding, and she saw a surgeon through her daughter, who was a nurse. “But several months, a few months later, it started up again, and that time, there was really a lot of bleeding. One day I went to the bathroom and there was really a lot of blood, and I started to have doubts. You know, we don’t know why, but we have a little… And my daughter, she works at the hospital, and she had referred me to a doctor, anyway, who… Well, I didn’t know him myself, but she said he was good, and I saw that he had a private clinic. I telephoned, and I made a appointment. So I went there on a Saturday morning, I went to see him one time, and he did an examination, and he said, ‘Ah, it looks like hemorrhoids, but I’d prefer to send you for a colonoscopy.” That surgeon saw her a few days later for a colonoscopy and then informed her of the diagnosis.

### The intervals

To calculate our pathways and define our time 0, we calculated the time elapsed between the first symptoms that elicited patients’ concern and the cancer diagnosis. These intervals ranged from one week to eight months. However, this variation was even greater (sometimes over a year) when taking into account symptoms identified by patients in the interviews that were potentially related to their cancer and had negatively affected their quality of life. In fact, patients often associated the first signs of colorectal cancer with the acute symptom phase. Given the nature of the investigation, in which the biopsy is performed at the same time as the colonoscopy if a lesion is found, and given that the cancer diagnosis is normally provided two or three days after the exam, we used the date of colonoscopy as the diagnosis date in calculating intervals.

### Explanatory factors related to colorectal cancer

The interviews revealed that patients sometimes had experienced signs suggestive of colorectal cancer over several years. However, these signs were interpreted in various ways (haemorrhoids, diabetes, hormonal disorders, abscess, mental illness). The symptoms’ non-specificity delayed investigation because neither the patients nor their physicians suspected colorectal cancer. In the case of colorectal cancer, the appraisal interval appeared particularly long.

### Factors related to patients

Because patients attributed their bleeding, weight loss, or fatigue to other causes, the investigation could occur years after the first signs. Often the patients initiated a consultation process but their physicians did not investigate them for colorectal cancer.

### Factors related to health system organization

Patients initiated consultation for signs suggestive of colorectal cancer very early, but the diagnostic process was such that colonoscopy was considered only after several other possible causes had been ruled out, thereby delaying the investigation. Often it was after symptoms had worsened that patients decided to re-consult their physician.

The patients in our sample appeared to have experienced significant delays in accessing colonoscopy, except for one who was hospitalized. Patients’ proactive attitudes helped them to surmount barriers to access to colonoscopy.

## Discussion

### Strengths and limitations of the study

We were able to estimate time intervals in the pre-diagnostic period, which is usually not possible for studies using cancer registries and administrative data [53]. As we selected three types of cancer, and as patients with breast cancer could be diagnosed because of symptoms or via the breast cancer screening program, we experienced a large variability in the characteristics of cancer trajectories. This variability is both a richness and a limitation. It allows us to better appraise the influence of the type of cancer on patients’ trajectories. For example, we saw that vague symptoms, such as for colorectal and lung cancer, made time intervals difficult to estimate as compared to breast cancer. Other elements, such as personal factors, appeared rather similar regardless of the type of cancer, as was also observed in the qualitative synthesis of Smith et al. (2005) [29]. Given that lung, breast, and colorectal cancers are the most commonly diagnosed, having this variety enables us to make recommendations for improving care with a potentially important impact for the population in our study context.

### Time elapsed before cancer diagnosis and determining factors

The first observation of note is that the intervals estimated from the interviews were different from those reported in the questionnaires. Intervals were particularly difficult to estimate for lung and colorectal cancer. Indeed, patients often had episodes of illness or discomfort (bronchitis, haemorrhoids, etc.) for which it was difficult to know whether cancer was the cause. In this interval between first symptoms and the initiation of investigation, the elapsed time was due primarily to patients’ personal attitudes, but also to the reactions of physicians, who did not at first suspect cancer [11]. Analyzing the experience of illness and of care from the patients’ perspective, as we did here, highlighted these aspects in ways that a retrospective analysis of medical charts could not [53, 54]. Our analysis indicated that it is difficult, if not impossible, to know exactly at what point the first symptoms should be attributed to cancer. The scientific literature attempts to divide the elapsed time into phases [7, 8, 10, 53, 55], but the appropriateness of that exercise is questionable because, even though each phase is part of the consultation process, both overall elapsed time and the various phases are concepts that are difficult to operationalize with any certainty.

Factors related to type of cancer explained many of the differences in the intervals. Our analyses concurred with those of Macleod et al. [11] and Smith et al. [29] in showing a direct correlation between atypical or vague symptoms and longer pre-diagnostic intervals. For colorectal cancer, the appraisal interval [7, 8, 10] could last several months or even years [12, 55]. Clearly, for colorectal cancers, there is a need to improve pre-diagnostic medical awareness, as symptoms are often not specific [14]. A breast mass, on the other hand, leaves little doubt about the potential existence of cancer, and this interval is quite short. For breast cancer, delays occur mainly in the help-seeking and investigation phases. Because of the acute nature of respiratory problems, the help-seeking interval in lung cancer cases is generally shorter [56]. When patients are shunted back into the appraisal phase, with the consequent delays, it is often because their physician did not initially attribute the symptoms to cancer. Thus, atypical symptoms will lead patients and clinicians to treat the symptoms and rule out other possible causes before investigating for cancer [11], leading to a looped process for the first three phases of the Model of Pathways to Treatment.

In terms of personal factors, even when patients are worried, they will tend to delay the decision to consult if their lives are very busy (work, vacation, family responsibilities) and the symptoms are not acute [55]. People with strong family or social support will receive more encouragement to visit a clinician [55], which may influence their decision to consult. Patients whose close family members or friends have experienced cancer are more likely to consult earlier. Some people delay investigation because they dread the technical procedure (mammography, colonoscopy) [29].

The interval between initiating investigation and reaching a diagnosis is most often explained by health system organization, mainly with regard to difficulties in accessing medical and diagnostic services and in the sequential investigation process. We also note that time to investigation is often shorter for patients going through the emergency room because of the simultaneous accessibility of a variety of medical specialties and technical platforms. This observation creates a dilemma for health system organization: whereas current primary care accessibility problems have repercussions that include treatment delays and inappropriate use of hospital emergency services [57], should patients confronted with these accessibility problems be encouraged to go to the emergency room when they experience symptoms they suspect are suggestive of cancer, at the risk of increasing emergency consultation demand for non-cancer related reasons? Several studies [58, 59] indicate that cancer diagnosis following emergency consultation results in poorer clinical outcomes, which may be explained by a more advanced stage of cancer. Emergency presentation as related to cancer is a complex phenomenon that is the result of various causes: no easy access to a GP, patients or GPs not recognizing the symptoms, difficult access to diagnostic and specialized resources and, of course, exacerbation of health status [59]. Murchie et al. [59] underscore the fact that emergency presentation “affords individual patients the best chance of rapid treatment and cure and does not always represent failure” (p. 10). In contexts where access to care—whether primary, diagnostic or specialized—is difficult, using the emergency room becomes important and legitimate and might improve clinical outcomes for patients.

### Avenues for improving early cancer diagnosis

One way to reduce delays in diagnosis is to address the interval between first symptoms and investigation. From an interventional perspective, an important question is how to make families [60], spouses [55], and clinicians [60] more alert to signs that could be suggestive of cancer. Would more awareness campaigns targeting the public and clinicians lead to more rapid reaction? (Tod and Joanne [61], in fact, developed an awareness-building tool for the NHS). Is it possible to reinforce families’ influence in triggering the consultation process? In their study, Austoker et al. [62] found that individual and community-based interventions to inform the public and encourage people to consult earlier for symptoms suggestive of cancer were not very effective. Finding the most effective way to reach the public and clinicians in order to foster early diagnosis is a major research challenge. The fact that each illness has its own natural evolution and elicits particular attitudes in patients toward signs and symptoms suggests that different strategies are needed for the different types of cancer [2].

It is also possible to change how care is organized. Several measures might be considered. First, in the 22 patients we met, we found two cases in which positive test results were not transmitted to the patients. It is true that we may have over-sampled for medical errors by purposefully selecting patients whose time to diagnosis was particularly long. Nevertheless, a mechanism should be developed to ensure this does not happen. Second, in cases of suspected cancer, it would be useful to provide integrated diagnostic services, where the technical platforms and a specialized team are together in one place, and where the patient could be seen by all relevant parties on the same day. This would, first, eliminate obstacles and shorten the wait for an appointment and, second, avoid the sequential investigation process. In fact, for breast cancer, the sequential investigation process that calls for the prescribing physician to also be the one who transmits the test results adds considerably to the investigation time. In contrast, when the investigation is concentrated in a single place and time (integrated process), the investigation process is very short. The three areas of intervention that we have highlighted—raising awareness among families and professionals and optimizing the diagnostic pathway—have also been raised by Molassiotis et al. [12] in the United Kingdom.

### Inequities in early cancer diagnosis

Our results raise important questions regarding equity of access. We found that expert patients, who had a good understanding of how the health system works and were well connected with health professionals, were able to shorten investigation times considerably. Several patients also opted to consult private clinics, at their own cost, to obtain diagnostic services more rapidly. These two points raise important questions in a universal healthcare system whose objective is to provide care based on need and not according to patients’ financial capacity or personal skills. Having either financial resources or a thorough knowledge of the healthcare system allows some patients to shorten the time intervals for a same physical condition. In such cases, access is not related to gravity of illness or care need, but rather to the capacity to mobilize one’s resources. The literature on early cancer diagnosis shows that socio-economically disadvantaged patients experience longer delays in obtaining care [11]. Our results indicate that such a situation might exist in Quebec, even in the presence of public and universal health coverage. In the case of Quebec, these issues could be resolved quickly if access to professional and diagnostic services were improved. In comparison with the rest of Canada and with OECD countries, Quebec’s performance in terms of access to diagnostic and specialized services is weak. In Quebec, 61% of physicians (as opposed to 38% in Canada) report that their patients often have difficulty obtaining specialized diagnostic tests [63], and patients tend to get around access barriers by using their own means.

## Conclusions

This study sheds light on why the elapsed time between first symptoms and cancer diagnosis is longer for some patients than for others. It also provides insight into the roles played by cancer type characteristics, personal attitudes, and health system organization. Numerous studies have delved into the behavioural and psychological processes associated with elapsed time that are attributable to patients [8, 29], but very few have analyzed the influence of these three factors [12]. Psychosocial and behavioural models, such as Andersen’s model and the Model of Pathways to Treatment [7, 8, 10], have introduced the influence of factors associated with cancer type and health system organization. However, our analysis showed that the links and interactions among these three types of factors are very close, making it difficult to apply such a model to represent the pathway leading to cancer diagnosis [64], especially for cancers whose signs and symptoms are vague and non-specific.

Quebec’s cancer registry has existed only since 2011. It compiles information on cancer mortality and incorporates increasingly more clinical information on stages of illness and on treatments provided. The registry does not contain information on primary care services received prior to diagnosis. We thus have very little information on the impediments to an optimal consultation and care pathway and on what happens before diagnosis. As such, this study sheds light on this poorly documented period and helps to identify measures that could be implemented for more timely diagnosis.

Our study revealed that there are inequities in access to medical and diagnostic services that could have consequences for early cancer diagnosis. This opens the way for a research agenda to document the scope of this phenomenon and identify solutions that would make universal and equitable access a reality in our healthcare system, and that would respect the core principles upon which Canada’s health system is founded.
